# Supplementary material for: Hybrid Models and Biological Model Reduction with PyDSTool
Source: PLoS Comput Biol. 2012 Aug 9;8(8):e1002628. doi: 10.1371/journal.pcbi.1002628 (PMC3415397; doi:10.1371/journal.pcbi.1002628)
Supplement: Text S4 — Complete source code for the PyDSTool package (version 0.88.120504). Includes API documentation and help files linking to web pages. This file is identical to the current public release on Sourceforge.net. (ZIP) [file pcbi.1002628.s004.zip › PyDSTool/html/PyDSTool.PyCont.TestFunc.BorderMethod-class.html]

xml version="1.0" encoding="ascii"?


PyDSTool.PyCont.TestFunc.BorderMethod


| Home | Trees | Indices | Help | PyDSTool |
| --- | --- | --- | --- | --- |

Package PyDSTool ::
Package PyCont ::
Module TestFunc ::
Class BorderMethod

[hide private]

[frames] | no frames]

# Class BorderMethod

source code

```
object --+        
         |        
  Function --+    
             |    
      TestFunc --+
                 |
                BorderMethod
```

Known Subclasses:
:   - Branch\_Bor
    - , Fold\_Bor
    - , Hopf\_Bor
    - , Hopf\_Double\_Bor\_One
    - , Hopf\_Double\_Bor\_Two

---

```
Border method:

    [A   B][V] = [0]
    [C^T D][G]   [1]

where r = corank and:
    A = (n,m)
    s = max(n,m)
    p = s-m+r, q = s-n+r
    B = (n,p)
    C = (m,q)
    D = 0_(q,p)
    V = (m,q)
    G = (p,q)
    0 = (n,q)
    1 = (q,q)

F:R^nm --> R^pq,  F(A) = G

It can also be written as:

    [W^T G][A   B] = [0 1]
           [C^T D]

where now:
    W = (n,p)
    0 = (p,m)
    1 = (p,p)

This is important for calculating derivatives:

    S_{z} = -W^T x A_{z} x V

This is implemented in the diff method.

In order to use the Function class, the matrices are put into vector form.
```


|  |  |
| --- | --- |
| Instance Methods | [hide private] |

|  |  |
| --- | --- |
| \_\_init\_\_(self, (a, b), (n, m), F, C, r=1, update=False, corr=False, save=False, numpoints=None)  F: R^a --> R^b | source code |

|  |  |
| --- | --- |
| setdata(self, A)  Note: p, q <= min(n,m) | source code |

|  |  |
| --- | --- |
| updatedata(self, A) | source code |

|  |  |
| --- | --- |
| func(self, A) | source code |

|  |  |
| --- | --- |
| getVW(self, A) | source code |

**Inherited from `TestFunc`**:
`diff`,
`findzero`

**Inherited from `Function`**:
`__call__`,
`__getitem__`,
`__setitem__`,
`numhess`

**Inherited from `object`**:
`__delattr__`,
`__getattribute__`,
`__hash__`,
`__new__`,
`__reduce__`,
`__reduce_ex__`,
`__repr__`,
`__setattr__`,
`__str__`


|  |  |
| --- | --- |
| Properties | [hide private] |

**Inherited from `object`**:
`__class__`


|  |  |
| --- | --- |
| Method Details | [hide private] |

|  |  |
| --- | --- |
| \_\_init\_\_(self, (a, b), (n, m), F, C, r=1, update=False, corr=False, save=False, numpoints=None)  *(Constructor)* | source code |

F: R^a --> R^b

Note: I did not assign b automatically (although I could - it would
just be b = p\*q) since you may not want to use all of the entries of S.
Some bordering methods are not minimally augmented systems and thus only
require certain entries of S.

Overrides: object.\_\_init\_\_

  


| Home | Trees | Indices | Help | PyDSTool |
| --- | --- | --- | --- | --- |

|  |  |
| --- | --- |
| Generated by Epydoc 3.0.1 on Fri May 4 15:24:07 2012 | http://epydoc.sourceforge.net |
